# Learning Promotes Subfield-Specific Synaptic Diversity in Hippocampal CA1 Neurons

**DOI:** 10.1093/cercor/bhz022

**Published:** 2019-02-23

**Authors:** Y Sakimoto, J Mizuno, H Kida, Y Kamiya, Y Ono, D Mitsushima

**Affiliations:** 1 Department of Physiology, Yamaguchi University Graduate School of Medicine, Yamaguchi, Japan; 2 Department of Physiology and Neuroscience, Kanagawa Dental University, Kanagawa, Japan; 3 The Research Institute for Time Studies, Yamaguchi University, Yamaguchi, Japan; 4 Uonuma Institute of Community Medicine, Niigata University Medical Hospital, Niigata, Japan; 5 Department of Electronics and Bioinformatics, Meiji University School of Science and Technology, Tokyo, Japan

**Keywords:** AMPA receptor, contextual learning, GABA_A_ receptor, self-entropy, synaptic plasticity

## Abstract

The hippocampus is functionally heterogeneous between the dorsal and ventral subfields with left–right asymmetry. To determine the possible location of contextual memory, we performed an inhibitory avoidance task to analyze synaptic plasticity using slice patch-clamp technique. The training bilaterally increased the AMPA/NMDA ratio at dorsal CA3–CA1 synapses, whereas the training did not affect the ratio at ventral CA3–CA1 synapses regardless of the hemisphere. Moreover, sequential recording of miniature excitatory postsynaptic currents and miniature inhibitory postsynaptic currents from the same CA1 neuron clearly showed learning-induced synaptic plasticity. In dorsal CA1 neurons, the training dramatically strengthened both excitatory and inhibitory postsynaptic responses in both hemispheres, whereas the training did not promote the plasticity in either hemisphere in ventral CA1 neurons. Nonstationary fluctuation analysis further revealed that the training bilaterally increased the number of AMPA or GABA_A_ receptor channels at dorsal CA1 synapses, but not at ventral CA1 synapses, suggesting functional heterogeneity of learning-induced receptor mobility. Finally, the performance clearly impaired by the bilateral microinjection of plasticity blockers in dorsal, but not ventral CA1 subfields, suggesting a crucial role for contextual learning. The quantification of synaptic diversity in specified CA1 subfields may help us to diagnose and evaluate cognitive disorders at the information level.

## Introduction

The hippocampus is functionally heterogeneous between the dorsal and ventral subfields (Fanselow and Dong 2010), with left–right asymmetry (Shinohara et al. 2013). Dorsal subfields seem to serve cognitive functions, whereas ventral subfields correspond to the affective hippocampus (Moser and Moser 1998). Moreover, the acquisition of some hippocampal-dependent tasks seems to require the left–right asymmetry of the hippocampal circuit (Goto et al. 2010). Using an inhibitory avoidance (IA) task with a hippocampus-dependent contextual learning paradigm (Izquierdo et al. 1998), we previously found that contextual learning requires synaptic plasticity for both excitatory and inhibitory inputs at CA1 synapses (Mitsushima et al. 2011, 2013). However, there is no synaptic evidence to prove the location of encoded memory within a broad CA1 area.

First, we analyzed learning-induced synaptic plasticity in 4 CA1 subfields to analyze the learning-induced synaptic plasticity. Second, we sequentially recorded miniature EPSCs (mEPSCs) and miniature IPSCs (mIPSCs) in the same neuron to specify the CA1 subfield of learning-created synaptic diversity. Considering that each presynaptic vesicle contains approximately 2000 glutamate (Ryan et al. 1993; Hori and Takahashi 2012) or 2500 GABA molecules (Telgkamp et al. 2004; Pugh and Raman 2005), the mE(I)PSC analysis allows for the quantification of postsynaptic currents and plasticity. Nonstationary fluctuation analysis further revealed subfield-specific evidence of learning at a single-channel level. Moreover, by analyzing the appearance probability of the synaptic strength in each neuron, we proposed a new approach to quantify learning-induced synaptic diversity as self-entropy increases after the training. The learning clearly increased the cell-specific self-entropy levels in dorsal, but not ventral CA1 subfields, and local blockade of the synaptic plasticity blocked the learning in dorsal but not ventral CA1 subfields, suggesting contributory CA1 subfields at the information level. Since learning is known to modulate both excitatory and inhibitory synaptic plasticity in key brain areas such as hippocampus (Mitsushima et al. 2013), amygdala (Lin et al. 2011; Ganea et al. 2015), or cortical areas (Ghosh et al. 2015, 2016; Kida et al. 2016), this approach may help us to diagnose and evaluate cognitive disorders in multiple brain regions.

## Materials and Methods

### Animals

Young male Sprague-Dawley rats (postnatal 28–31 days of age) were used. After weaning, same sex groups of 2–3 rats were housed in plastic cages (length 25 cm, width 40 cm, height 25 cm) at a constant temperature of 23 ± 1 °C under a constant cycle of light and dark (light on: 8:00 A.M. to 8:00 P.M.). But, the rats were individually housed at least 24 h prior to the experiment to avoid any episodic experience. Food (MF, Oriental Yeast Co. Ltd, Tokyo Japan) and tap water were available ad libitum in all experimental periods. All animal housing and surgical procedures followed the guidelines of the Institutional Animal Care and Use Committee of Kanagawa Dental University and Yamaguchi University. The guidelines comply with the Guide for the Care and Use of Laboratory Animals published by the National Institute of Health (NIH Publication No. 85-23, revised 1996).

### Inhibitory Avoidance Task

**Figure 1.**
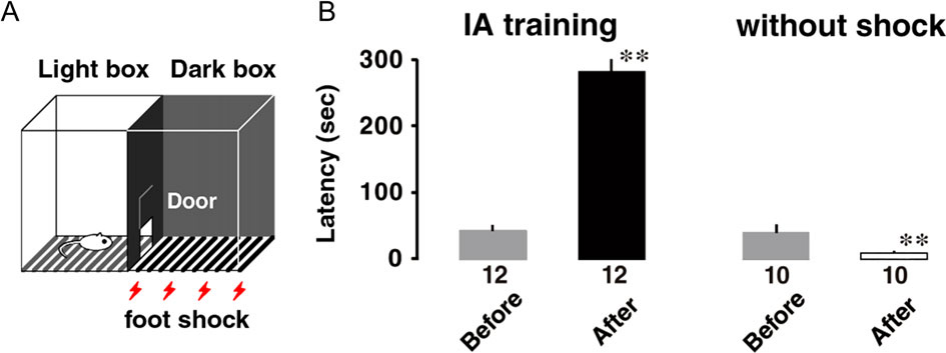
Inhibitory avoidance (IA) task. (*A*) IA training apparatus. (*B*) Foot-shock increased the latency to entering the dark box. The number of rats in each group is shown at the bottom of each bar. Error bars indicate + standard error of the mean (SEM). **P < 0.01 versus before the training.

The IA training apparatus (length: 33 cm, width: 58 cm, height: 33 cm) was a 2-chambered box consisting of a lighted safe side and a dark shock side separated by a trap door (Fig. 1*A*; Mitsushima et al. 2011, 2013). For training, rats were placed in the light side of the box facing a corner opposite the door. After the trap door was opened, the rats could enter the dark box at will. The latency before entering the novel dark box was measured as a behavioral parameter (latency before IA learning). Soon after the animals entered the dark side, we closed the door and applied a scrambled electrical foot-shock (2 s, 1.6 mA) via electrified steel rods in the floor of the box. The rats were kept in the dark compartment for 10 s before being returned to their home cage. Untrained control rats were not moved from their home cages.

Thirty minutes after the procedure described above, the rats were placed in the light side. The latency before entering the dark box was measured as an indicator of learning performance (latency after IA learning).

### Drug Injection

Under sodium pentobarbital anesthesia (30–50 mg/kg, i.p.), a stainless-steel guide cannula (outer diameter, 0.51 mm) was implanted stereotaxically into the just above the target region of the dorsal or ventral hippocampus. The experiment was performed 1–3 days after the implantation. After cannula implantation, a stylet was inserted into the guide until drug injection.

On the day of the experiment, the stylet was replaced with 1.0 mm longer injector without restraint animals in their home cage (outer diameter 0.31 mm). The coordinates of dorsal CA1 were 3.0 mm posterior to bregma, 2.0 mm lateral to the midline, and 3.8 mm below the surface of the skull. The coordinates of ventral CA1 were 4.2 mm posterior to bregma, 5.5 mm lateral to the midline, and 5.5 mm below the surface of the skull.

Approximately 20 min before the IA learning procedure, saline, NMDA receptor antagonist (3 μg/μL per side, d-AP5, Sigma Chemical Co., St. Louis, MO), or nicotinic α_7_ receptor antagonist (40 μg/μL per side, methyllycaconitine citrate, Sigma) was directly injected into the CA1 through fine flexible silicone tubing (o.d. 0.5 mm, Kaneka Medix Co. Osaka, Japan) without restraining the animals. We used AP5 to block AMPA receptor-mediated plasticity at excitatory synapses (Morris et al. 1986; Park et al. 2004), and also used Mla to block GABA_A_ receptor-mediated plasticity at inhibitory synapses. Mla is known to block acetylcholine-induced strengthen of GABA_A_ receptor-mediated postsynaptic current in CA1 pyramidal neurons (Mitsushima et al. 2013; Townsend et al. 2016).

### Electrophysiological Recordings

We recently published detailed technical protocol of slice-patch clamp technique for analyzing learning-induced synaptic plasticity with a short demonstration movie (Kida et al. 2017). Using the technique, we examined learning-induced synaptic plasticity in dorsal or ventral CA1 neurons.

One hour after the paired foot-shock, rats were anesthetized with pentobarbital and acute brain slices prepared (Mitsushima et al. 2011, 2013). We used naïve rats for untrained group, which were injected with the same dose of anesthesia in their home cage. The results in unpaired or walk-through controls were reported previously (Mitsushima et al. 2013). For the whole-cell recordings (Kida et al. 2016), the brains were quickly perfused with ice-cold dissection buffer (25.0 mM NaHCO_3_, 1.25 mM NaH_2_PO_4_, 2.5 mM KCl, 0.5 mM CaCl_2_, 7.0 mM MgCl_2_, 25.0 mM glucose, 90 mM choline chloride, 11.6 mM ascorbic acid, 3.1 mM pyruvic acid) and gassed with 5% CO_2_/95% O_2_. Coronal (target CA1 area: AP −3.8 mm, DV 2.5 mm, LM ± 2.0 mm) or horizontal brain slices (target CA1 area: AP −5.2 mm, DV 7.7 mm, LM ± 5.8 mm) were cut (350 μm, Leica vibratome, VT-1200) in dissection buffer and transferred to physiological solution (22–25 °C, 114.6 mM NaCl, 2.5 mM KCl, 26 mM NaHCO_3_, 1 mM NaH_2_PO_4_, 10 mM glucose, 4 mM MgCl_2_, 4 mM CaCl_2_, pH 7.4, gassed with 5% CO_2_/95% O_2_). The recording chamber was perfused with physiological solution containing 0.1 mM picrotoxin and 4 μM 2-chloroadenosine at 22–25 °C. For the mEPSC and mIPSC recordings, we used the physiological solution containing 0.5 μM TTX to block Na^+^ channels.

Patch recording pipettes (4–7 MΩ) were filled with intracellular solution (127.5 mM cesium methanesulfonate, 7.5 mM CsCl, 10 mM Hepes, 2.5 mM MgCl_2_, 4 mM Na_2_ATP, 0.4 mM Na_3_GTP, 10 mM sodium phosphocreatine, 0.6 mM EGTA at pH 7.25). Whole-cell recordings were obtained from CA1 pyramidal neurons from the rat hippocampus using an Axopatch 700 A amplifier (Axon Instruments). The whole-cell patch-clamp data were collected with Clampex 10.4, and the data were analyzed using Clampfit 10.4 software (Axon Instruments).

### The AMPA/NMDA Ratio

The AMPA/NMDA ratio is conventional way to evaluate postsynaptic plasticity at glutamatergic excitatory synapses. Since concomitant increases in both components may not change the ratio, further analysis of AMPA evoked responses is necessary to elucidate the receptor-specific plasticity (i.e., *I*/*O* curve for evoked EPSCs, amplitude of miniature AMPA receptor-mediated current, or further fluctuation analysis of the current). The recording chamber was perfused with physiological solution bubbled with the gas mixture and maintain the temperature at 22–25 °C. Then, we added 0.1 mM picrotoxin to the solution to block the GABA_A_-mediated response. We also added 4 μM 2-chloroadenosine to stabilize the evoked neural response. The patch recording pipettes were filled with the intracellular solution for voltage-clamp recordings. The resistance of the recording pipette in the aCSF was between 4 and 7 MΩ.

To analyze the function of CA3–CA1 synapses, bipolar tungsten stimulating electrodes (Unique Medical Co., Ltd., Tokyo, Japan) were placed in CA1 ~200–300 μm lateral from recorded cells. The electrically evoked EPSC amplitudes were measured from the peak of the postsynaptic current to the basal current level immediately before electrical stimulation. The stimulus intensity was increased until a synaptic response with an amplitude >−10 pA was recorded. AMPA/NMDA ratios were calculated as the ratio of the peak current at −60 mV to the current at +40 mV 150 ms after stimulus onset (40–60 traces averaged for each holding potential).

### Miniature Postsynaptic Current Recordings

Miniature excitatory postsynaptic currents (mEPSCs) are thought to correspond to the responses elicited by the presynaptic release of a single vesicle of glutamate. In contrast, miniature inhibitory postsynaptic currents (mIPSCs) are thought to correspond of GABA. Increase in the amplitudes of mEPSCs and mIPSCs reflect postsynaptic transmission strengthening, while increase in the event frequency reflects increases in the number of functional synapses or the presynaptic release probability.

For the miniature recordings, the mEPSCs (−60 mV holding potential) and mIPSCs (0 mV holding potential) were recorded sequentially for 5 min in the same CA1 neuron. The miniature events were detected using the software, and the events above 10 pA were used for the analysis. We recorded at least for 5 min to determine the events frequency of mEPSCs or mIPSCs. The amplitudes of the events were averaged to obtain the mean amplitude. Bath application of an AMPA receptor blocker (CNQX, 10 μM) or GABA_A_ receptor blocker (bicuculline methiodide, 10 μM) consistently blocked the mEPSC or mIPSC events, respectively.

### Nonstationary Fluctuation Analysis

AMPA receptor-mediated evoked EPSCs and GABA_A_ receptor-mediated mIPSCs were analyzed by nonstationary fluctuation analysis (Ghosh et al. 2015; Ono et al. 2016). To isolate fluctuations in current decay due to stochastic channel gating, the mean waveform was scaled to the peak of individual E(I)PSCs. The requirements for such analysis include a stable current decay time course throughout the recording and an absence of any correlation between the decay time course and peak amplitude. The relationship between the peak-scaled variance and the mean current is given by the following equation:

$$\sigma^{2}=iI-I^{2}/N+b_{l}$$

where *σ*^2^ is the variance, *I* is the mean current, *N* is the number of channels activated at the peak of the mean current, *i* is the unitary conductance, and *b*_*l*_ is the background variance. In our experiments, 31–69 EPSCs and 14–133 IPSCs were analyzed from selected epochs in each of the cells in which there was no correlation between current decay (63% decay time) and peak amplitude (*P* > 0.05, Spearman’s rank-order correlation test). The weighted mean channel current can be estimated by fitting the full parabola or initial slope of the relationship. All the analysis was done using MATLAB software (MathWorks, MA, USA). The number of channels was further divided by the corresponding value of mean E(I)PSC amplitude to obtain the single channel current.

### Self-Entropy Analysis

We used standard spreadsheet software (Excel 2010, Microsoft Co., Redmond, WA, USA) to calculate the self-entropy per neuron. First, we obtained 4 miniature parameters (mean mEPSC amplitude, mean mIPSC amplitude, mean mEPSC frequency, and mean mIPSC frequency) in individual CA1 pyramidal neurons. Then, we determined the distribution of appearance probability of 4 miniature parameters separately using 1-dimensional Kernel density analysis. Geometric/topographic feature of the appearance probability was calculated using Kernel density analysis. Let *X*_1_, *X*_2_,…, *X*_*n*_ denote a sample of size *n* from real observations. The Kernel density estimate of *P* at the point *x* is given by the following equations:

$$P_{n}\left(x\right)=\frac{1}{nh}\overset{n}{\underset{i=1}{\sum }}K\left(\frac{x-X_{i}}{h}\right)$$

where *K* is a smooth function called the Gaussian kernel function and *h* > 0 is the smoothing bandwidth that controls the amount of smoothing. We chose Silverman’s reference bandwidth or Silverman’s rule of thumb (Silverman 1986; Sheather 2004). It is given by the following equation:

$$h\;=\;0.9An^{-1/5}$$

where *A* = min (standard deviation, interquartile range/1.34). By normalizing integral value in untrained controls, we found the distribution of appearance probability at any point. Then, we calculated the appearance probability at selected points. All data points for probability in untrained and trained rats were converted to self-entropy (bit) using the Shannon entropy concept, defined from the Information Theory (Shannon 1948).

To calculate using spreadsheet software, the data of 4 miniature parameters were summarized in 4 different sheet, and we obtained the bandwidth (*h*) of individual parameter in untrained group using a formula [=0.9 STDEV (neuron 1, neuron 2,,, neuron *N*)/COUNT (neuron 1, neuron 2,,, neuron *N*) ^ (1/5)]. Then, using the data of untrained group, we calculated the distribution of appearance probability as follows:

- Probability distribution of first data of a parameter (neuron 1) was calculated using a formula [=EXP(−(((data of neuron 1 − any point)/*h*)^2/2))/SQRT (2 * PI())].
- Also, probability distribution of second data of the parameter (neuron 2) was calculated using the formula [=EXP(−(((data of neuron 2 − any point)/*h*)^2/2))/SQRT(2 * PI())].
- Similarly, probability distribution of *N* data of the parameter (neuron *N*) was calculated using the formula [=EXP(−(((data of neuron *N* − any point)/*h*)^2/2))/SQRT(2 * PI())].
- Sum all probability distribution from neuron 1 to *N*, and the integral value was normalized to 1.

Based on the probability distribution, we calculated individual appearance probability of all recorded neurons. Then, the appearance probability of the neuron was converted to the self-entropy using Shannon’s formula (= −LOG [appearance probability of the neuron, 2]) (Fig. 3*F*). For graphic expression, the distribution was visualized 2-dimensionally in the R software environment (R Foundation for Statistical Computing, Vienna, Austria) (Figs 3*C*,*G*,*L*,*P* and 4*B*,*F*,*J*,*N*).

### Statistical Analysis

We used the paired *t* test to analyze IA latency and unpaired *t* test to analyze estimated open channel numbers. The AMPA/NMDA ratio, mEPSC, mIPSC, and self-entropy were analyzed using 2-way factorial ANOVA in which the between-group factors were laterality and training. We used one-way factorial ANOVA to evaluate the difference in miniature responses between dorsal and ventral synapses. The Shapiro–Wilk test and *F*-test were used for normality and equality of variance, respectively. Because the self-entropy data had large variations within a group, we performed log (1 + *x*) transformation prior to the analysis (Mitsushima et al. 1994). *P* < 0.05 was considered significant.

## Results

### Inhibitory Avoidance Task

To investigate a possible location of the contextual memory in 4 CA1 subfields, rats were subjected to an IA task (Fig. 1*A*; Izquierdo et al. 1998; Mitsushima et al. 2011, 2013). In this learning paradigm, rats were allowed to cross from a light box to a dark box, where an electric foot-shock (1.6 mA, 2 s) was delivered. Half an hour after the IA task, we measured the latency in the illuminated box as contextual learning performance. With paired foot-shock, the latency was much longer after training than before the training (*t*_11_ = 14.0, *P* < 0.0001).

### AMPA/NMDA Ratio

To specify the subfields where the contextual learning drives AMPA receptors into CA3–CA1 synapses, we measured the AMPA- to NMDA-type glutamate receptor response ratio in the dorsal or ventral hippocampus of both hemispheres.

**Figure 2.**
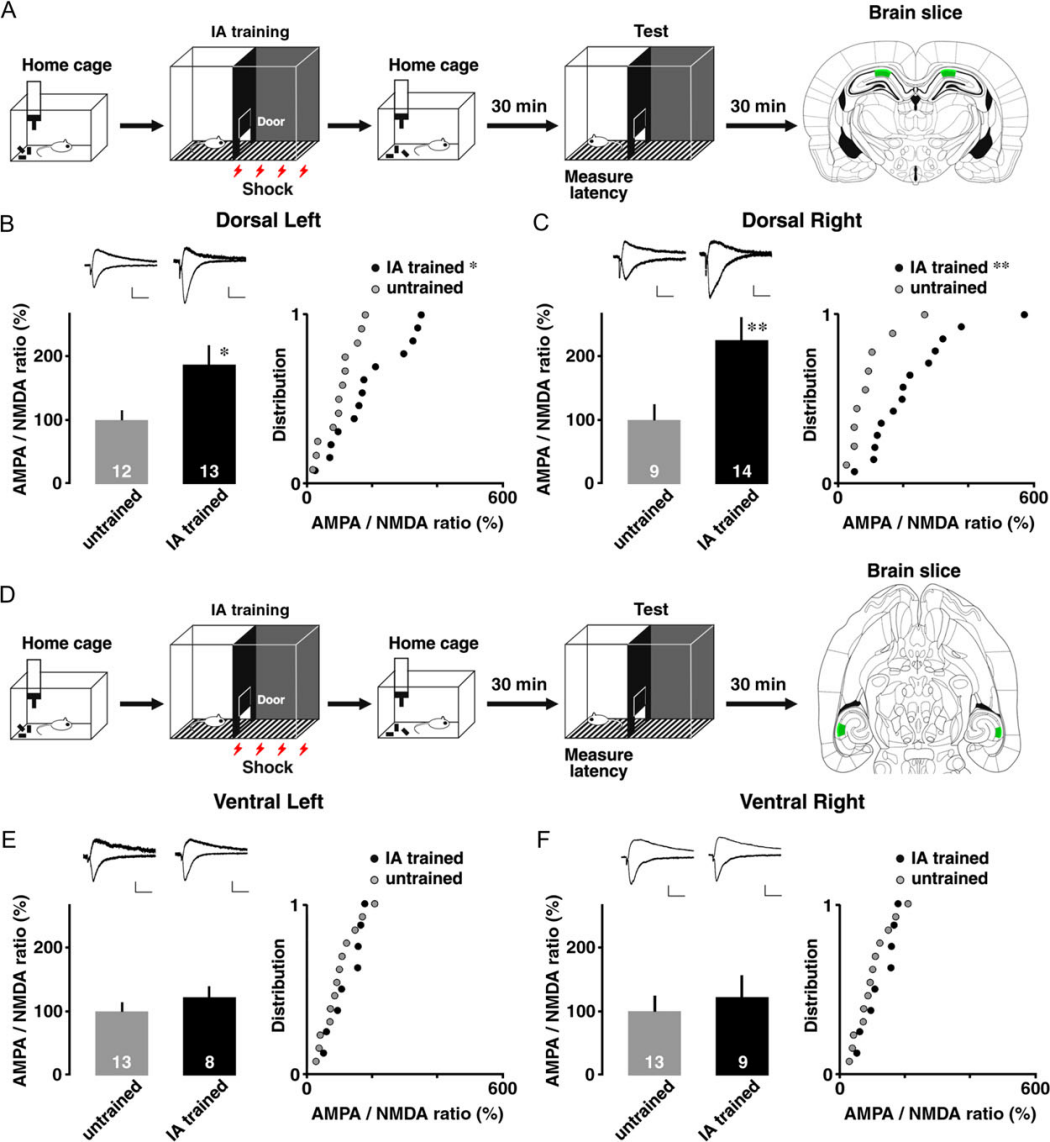
AMPA/NMDA ratio in 4 CA1 subfields. (*A*) Experimental design and coronal section of dorsal CA1 for patch-clamp analysis. (*B*) The AMPA/NMDA ratio and cumulative distribution in dorsal left and (*C*) right CA1 neurons. The trained rats had significantly higher ratios at dorsal CA3–CA1 synapses than untrained rats. (*D*) Experimental design and horizontal section of ventral CA1 for patch-clamp analysis. (*E*) The AMPA/NMDA ratio and distribution in ventral left and (*F*) right CA1 neurons. IA training did not affect the ratio of ventral CA3–CA1 synapses. Green squares indicate the recorded CA1 subfields in the dorsal and ventral hippocampus. Upper insets show representative traces. The number of cells in each group is shown at the bottom of each bar. Vertical bar = 40 pA; horizontal bar = 50 ms. Error bars indicate + SEM. *P < 0.05, **P < 0.01 versus untrained.

Figure 2*A* shows experimental design and the recording location of dorsal CA1 neurons. At dorsal CA3–CA1 synapses, 2-way ANOVA revealed a significant main effect of training (*F*_1,44_ = 12.637, *P* = 0.0009), but the main effect of laterality (*F*_1,44_ = 0.007, *P* = 0.93) or interaction (*F*_1,44_ = 0.156, *P* = 0.70) was not significant (Fig. 2*B*,*C*). Figure 2*D* shows experimental design and the recording location of ventral CA1 neurons. At ventral CA3–CA1 synapses, the main effects of training (*F*_1,39_ = 0.960, *P* = 0.22), laterality (*F*_1,39_ = 1.641, *P* = 0.21), and interaction (*F*_1,39_ = 0.022, *P* = 0.88) were not significant (Fig. 2*E*,*F*). These results suggest that the training bilaterally strengthened AMPA receptor-mediated CA3–CA1 synapses in dorsal CA1 neurons, regardless of the hemisphere, but not in ventral CA1 neurons.

### Miniature Postsynaptic Currents in Dorsal CA1 Neurons

**Figure 3.**
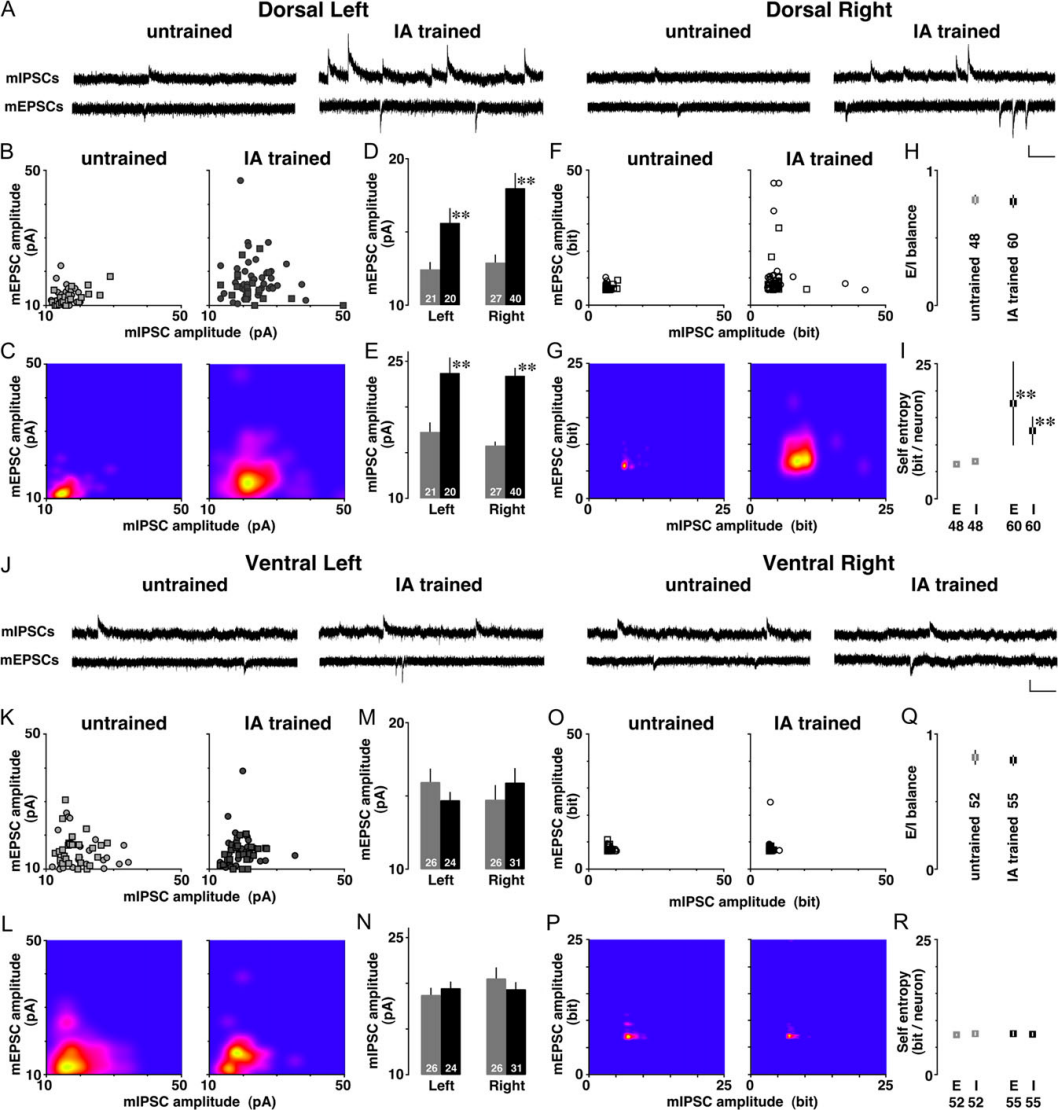
Diversity of mEPSC/mIPSC amplitudes and self-entropy per neuron after training. (*A*) Representative traces of mEPSCs and mIPSCs in dorsal left and right CA1 neurons. mEPSCs and mIPSCs were sequentially recorded from the same CA1 neuron in the presence of tetrodotoxin (0.5 μM). (*B*) 2-Dimensional plot of the amplitudes of mean mEPSC and mIPSC. The circle or square plot indicates the data from a right or left CA1 neuron, respectively. (*C*) Kernel density analysis visualized the distribution of appearance probability at any point. (*D*) Mean amplitudes of mEPSCs and (*E*) mIPSCs in dorsal CA1 neurons. IA training significantly increased both amplitudes in both hemispheres. (*F*) The self-entropy of each dot and (*G*) visualized density in dorsal CA1 neurons. (*H*) E/I balance of miniature amplitudes and (*I*) mean self-entropy per dorsal CA1 neuron. (*J*) Representative traces in ventral left and right CA1 neurons. (*K*) 2-Dimensional plot of the mean mEPSC and mIPSC amplitudes in ventral CA1 neurons and (*L*) and visualized distribution of appearance probability at any point. (*M*) Mean amplitudes of the mEPSCs and (*N*) mIPSCs in ventral CA1 neurons. IA training affected neither of them, regardless of the hemisphere. (*O*) The self-entropy of each dot and (*P*) visualized density in ventral CA1 neurons. (*Q*) E/I balance of miniature amplitudes and (*R*) mean self-entropy per ventral CA1 neuron. E = mEPSC; I = mIPSC, vertical bar = 20 pA; horizontal bar = 200 ms. Gray indicates untrained groups and black is trained groups. The number of cells in each group is shown at the bottom of each bar. Error bars indicate ± SEM. *P < 0.05, **P < 0.01 versus untrained.

To further analyze the learning-dependent synaptic plasticity, we recorded mEPSC or mIPSC in the presence of 0.5 μM TTX on both sides of the dorsal hippocampus (Fig. 3*A*). By changing the membrane potential, we sequentially recorded mEPSCs (at −60 mV) and mIPSCs (at 0 mV) from the same neuron as reported previously (Mitsushima et al. 2013). The postsynaptic currents are thought to correspond to the response elicited by a single vesicle of glutamate or GABA. In contrast, the number of synapses is known to affect the frequency of the events (Pinheiro and Mulle 2008).

At dorsal CA1 synapses, the strength of AMPA receptor-mediated excitatory inputs versus GABA_A_ receptor-mediated inhibitory inputs was measured in each neuron and plotted 2-dimensionally (Fig. 3*B*). The Kernel analysis revealed the distribution of appearance probability (Fig. 3*C*). Although untrained rats exhibited low and narrow distribution range, IA-trained rats had a broad distribution suggesting a diversity of synaptic input onto CA1 neurons. For mEPSCs, 2-way ANOVA revealed a significant main effect of training (*F*_1,104_ = 18.780, *P* < 0.0001), but the main effect of laterality (*F*_1,104_ = 2.237, *P* = 0.14) or interaction (*F*_1,104_ = 0.998, *P* = 0.32) was not significant (Fig. 3*D*). For mIPSCs, 2-way ANOVA revealed a significant main effect of training (*F*_1,104_ = 44.627, *P* < 0.0001), but the main effect of laterality (*F*_1,104_ = 0.724, *P* = 0.40) or interaction (*F*_1,104_ = 0.299, *P* = 0.59) was not significant (Fig. 3*E*). These results suggest that the training bilaterally strengthened both excitatory and inhibitory synapses onto dorsal CA1 neurons, regardless of the hemisphere.

The balance of excitatory/inhibitory (*E*/*I*) inputs was obtained by dividing the mean mEPSC amplitude by the mean mIPSC amplitude of the same neuron. For the *E*/*I* balance of miniature amplitudes, the main effect of training (*F*_1,104_ = 0.203, *P* = 0.65), laterality (*F*_1,104_ = 2.469, *P* = 0.12), or interaction (*F*_1,104_ = 0.002, *P* = 0.96) was not significant (Fig. 3*H*). Thus, the training did not affect the balance of mEPSC versus mIPSC amplitudes, suggesting the balance of excitatory versus inhibitory input strength onto dorsal CA1 neurons.

### Self-Entropy in Dorsal CA1 Neurons

Based on the information theory of Shannon (1948), we calculated appearance probability of the mean amplitudes of mEPSCs and mIPSCs. First, we found the distribution of appearance probability in untrained controls (Fig 3*C*, left), and then we analyzed the appearance probability of all recorded neurons one-by-one. Each probability of single neuron was calculated as the self-entropy and plotted 2-dimensionally (Fig. 3*F*). For example, a point with high appearance probability (around the mean level of mE(I)PSC amplitude) indicates low self-entropy, whereas a point with very rare probability (a deviated point of mE(I)PSC amplitude) indicates high self-entropy.

We found that all recorded neurons exhibited different self-entropy each other (Fig. 3*F*). In the dorsal CA1, self-entropy in the excitatory synapse exhibited a significant main effect of training (*F*_1,104_ = 9.322, *P* = 0.0029), but the main effect of laterality (*F*_1,104_ = 1.229, *P* = 0.27) or interaction (*F*_1,104_ = 0.879, *P* = 0.35) was not significant (Fig. 3*F*). Similarly, self-entropy in the inhibitory synapse exhibited a significant main effect of training (*F*_1,104_ = 21.393, *P* < 0.0001), but the main effect of laterality (*F*_1,104_ = 1.205, *P* = 0.27) or interaction (*F*_1,104_ = 0.007, *P* = 0.93) was not significant (Fig. 3*F*). The Kernel analysis further visualized the density distribution (Fig. 3*G*). Thus, the training clearly increased the self-entropy of dorsal CA1 neurons in both hemispheres. The average level was 13.4 ± 0.2 bits in untrained rats, whereas IA-trained rats showed 30.3 ± 8.0 bits per single CA1 neuron (Fig. 3*I*).

### Miniature Postsynaptic Currents in Ventral CA1 Neurons

Conversely in ventral CA1 neurons, IA training did not affect the miniature responses (Fig. 3*K*). For mEPSCs, the main effects of training (*F*_1,103_ = 0.002, *P* = 0.96), laterality (*F*_1,103_ = 0.0002, *P* = 0.99), and interaction (*F*_1,103_ = 1.748, *P* = 0.19) were not significant (Fig. 3*M*). For mIPSCs, the main effects of training (*F*_1,103_ = 0.052, *P* = 0.82), laterality (*F*_1,103_ = 0.833, *P* = 0.36), and interaction (*F*_1,103_ = 1.025, *P* = 0.31) were not significant (Fig. 3*N*). The Kernel analysis visualized the distribution of appearance probability (Fig. 3*L*). Thus, the training did not affect the amplitudes in either hemisphere. These results suggest that the training strengthened neither excitatory nor inhibitory synapses onto ventral CA1 neurons, regardless of the hemisphere.

For the E/I balance of miniature amplitudes, the main effects of training (*F*_1,103_ = 0.244, *P* = 0.62), laterality (*F*_1,103_ = 0.069, *P* = 0.79), and interaction (*F*_1,103_ = 2.287, *P* = 0.13) were not significant (Fig. 3*Q*). The training did not affect the balance of mEPSC versus mIPSC amplitudes, suggesting the balance of excitatory versus inhibitory input strength onto ventral CA1 neurons.

### Self-Entropy in Ventral CA1 Neurons

Using the distribution of appearance probability in untrained controls (Fig 3*L*, left), we calculated the self-entropy of all recorded neurons one-by-one (Fig. 3*O*). We found all recorded neurons exhibited different self-entropy each other. In ventral CA1 neurons, self-entropy in the excitatory synapse did not exhibit a significant main effect of training (*F*_1,103_ = 0.001, *P* = 0.97), laterality (*F*_1,103_ = 0.356, *P* = 0.55), or interaction (*F*_1,103_ = 0.930, *P* = 0.34). Similarly, self-entropy in the inhibitory synapse did not exhibit a significant main effect of training (*F*_1,103_ = 1.284, *P* = 0.26), laterality (*F*_1,103_ = 1.158, *P* = 0.28), or interaction (*F*_1,103_ = 2.023, *P* = 0.16). Thus, the training did not affect the self-entropy in either hemisphere, and the visualized density distribution was shown in Figure 3*P*. The average levels of self-entropy were 15.0 ± 0.2 bits (untrained) and 14.9 ± 0.3 bits (IA-trained) per single CA1 neuron (Fig. 3*R*).

### Frequencies of the mE(I)PSC Events in Dorsal CA1 Neurons

**Figure 4.**
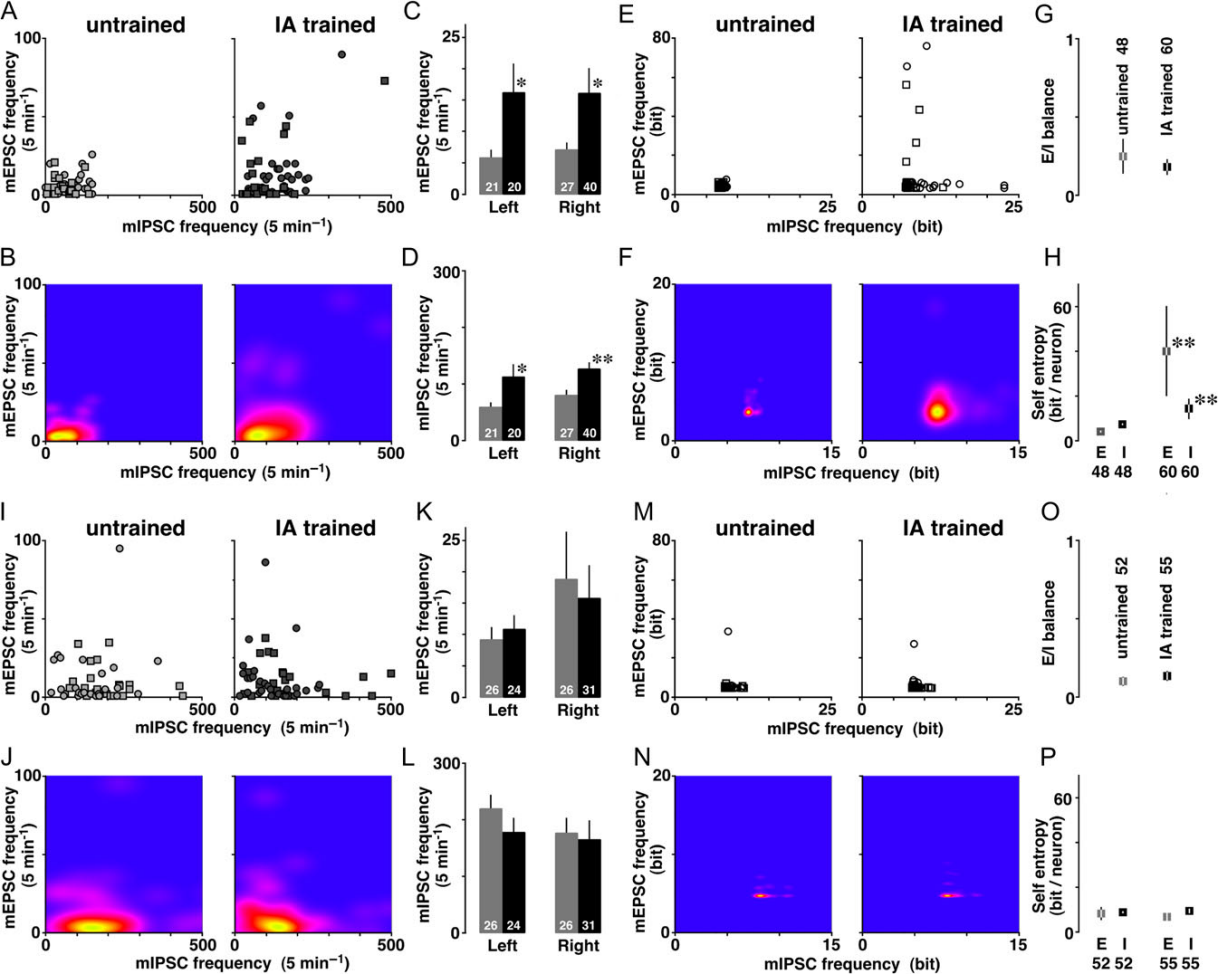
Diversity of mEPSC/mIPSC frequency and self-entropy per neuron after training. (*A*) 2-Dimensional plot of the frequency of mEPSCs and mIPSCs in dorsal CA1 neurons. The circle or square plot indicates the data from a right or left CA1 neuron, respectively. (*B*) Kernel density analysis visualized the distribution of appearance probability. (*C*) Mean frequencies of mEPSCs and (*D*) mIPSCs in dorsal CA1 neurons. IA training significantly increased both frequencies in both hemispheres. (*E*) The self-entropy of each dot and (*F*) visualized density in dorsal CA1 neurons. (*G*) E/I balance of miniature frequencies and (*H*) mean self-entropy per dorsal CA1 neuron. (*I*) 2-Dimensional plot of the self-entropy of the frequencies in ventral CA1 neurons. The circle or square plot indicates the data from right or left CA1 neuron, respectively. (*J*) Kernel density analysis visualized the distribution of appearance probability. (*K*) Mean frequencies of mEPSCs and (*L*) mIPSCs in ventral CA1 neurons. Right side exhibited significantly wider variation of mEPSC frequency than left side. IA training affected neither of them, regardless of the hemisphere. (*M*) The self-entropy of each dot and (*N*) visualized density in ventral CA1 neurons. (*O*) *E*/*I* balance of miniature frequencies and (*P*) mean self-entropy per ventral CA1 neuron. *E* = mEPSC; *I* = mIPSC. Gray indicates untrained groups and black is trained groups. The number of cells in each group is shown at the bottom of each bar. Error bars indicate ± SEM. *P < 0.05, **P < 0.01 versus untrained.

The number of functional synapses is known to affect the frequency of the mEPSC/mIPSC events. At dorsal CA1 synapses, the frequency of mEPSC versus mIPSC events was measured in each neuron and plotted 2-dimensionally (Fig. 4*A*). The Kernel analysis revealed the distribution of appearance probability (Fig. 4*B*). Although untrained rats exhibited low and narrow distribution range, IA-trained rats had a broad distribution suggesting a diversity of the number of functional synapses onto a single CA1 neuron. For mEPSCs, 2-way ANOVA revealed a significant main effect of training (*F*_1,104_ = 6.942, *P* = 0.0097), but the main effect of laterality (*F*_1,104_ = 0.023, *P* = 0.88) or interaction (*F*_1,104_ = 0.035, *P* = 0.85) was not significant (Fig. 4*C*). For mIPSCs, 2-way ANOVA revealed a significant main effect of training (*F*_1,104_ = 13.893, *P* = 0.0003), but the main effect of laterality (*F*_1,104_ = 1.760, *P* = 0.19) or interaction (*F*_1,104_ = 0.054, *P* = 0.82) was not significant (Fig. 4*D*). These results suggest that the training increased in the number of excitatory and inhibitory synapses onto dorsal CA1 neurons in both hemispheres.

The balance of excitatory/inhibitory (*E*/*I*) frequency was obtained by dividing the mean mEPSC frequency by the mean mIPSC frequency of the same neuron. For the *E*/*I* balance of miniature frequency, the main effect of training (*F*_1,104_ = 0.198, *P* = 0.66), laterality (*F*_1,104_ = 0.149, *P* = 0.70), or interaction (*F*_1,104_ = 0.78, *P* = 0.38) was not significant (Fig. 4*G*). Thus, the training did not affect the balance of mEPSC versus mIPSC frequency, suggesting the balance of the number of excitatory versus inhibitory synapses onto dorsal CA1 neurons.

### Self-Entropy of the Frequency in Dorsal CA1 Neurons

Using the distribution appearance probability in untrained controls (Fig. 4*B*, left), we analyzed the appearance probability at selected points. The probabilities at all data points were calculated as the self-entropy and plotted 2-dimensionally (Fig. 4*E*). Although all recorded neurons exhibited different self-entropy each other, the Kernel analysis further visualized the density distribution (Fig. 4*F*). IA training dramatically increased the amount of information per dorsal CA1 neurons (Fig. 4*H*).

Self-entropy in the mEPSC frequency exhibited a significant main effect of training (*F*_1,104_ = 7.944, *P* = 0.0058), but the main effect of laterality (*F*_1,104_ = 0.019, *P* = 0.89) or interaction (*F*_1,104_ = 0.067, *P* = 0.80) was not significant (Fig. 4*E*). Similarly, self-entropy in the mIPSC frequency exhibited a significant main effect of training (*F*_1,104_ = 6.753, *P* = 0.0107), but the main effect of laterality (*F*_1,104_ = 0.061, *P* = 0.81) or interaction (*F*_1,104_ = 0.001, *P* = 0.97) was not significant (Fig. 4*E*). Thus, the training clearly increased the self-entropy of dorsal CA1 neurons in both hemispheres, and the Kernel analysis further visualized the density distribution (Fig. 4*F*). The average level was 11.5 ± 0.2 bits in untrained rats, whereas the trained rats showed 54.5 ± 21.4 bits per single CA1 neuron (Fig. 4*H*).

### Frequencies of the mE(I)PSC Events in Ventral CA1 Neurons

IA training did not affect the frequency in ventral CA1 neurons. The frequency of mEPSC versus mIPSC events was measured in each neuron and plotted 2-dimensionally (Fig. 4*I*). Ventral CA1 neurons exhibited relatively wide distribution range in both untrained and IA-trained rats, and the Kernel analysis showed the distribution of appearance probability (Fig. 4*J*). For mEPSCs, the main effects of training (*F*_1,103_ = 0.017, *P* = 0.90), laterality (*F*_1,103_ = 2.144, *P* = 0.15), and interaction (*F*_1,103_ = 0.22, *P* = 0.64) were not significant (Fig. 4*K*). For mIPSCs, the main effects of training (*F*_1,103_ = 0.866, *P* = 0.35), laterality (*F*_1,103_ = 0.922, *P* = 0.34), and interaction (*F*_1,103_ = 0.273, *P* = 0.60) were not significant (Fig. 4*L*). Thus, the training affected frequency of neither mEPSC nor mIPSC regardless of the hemispheres. These results suggest that the training did not affect the number of excitatory and inhibitory synapses onto ventral CA1 neurons, regardless of the hemisphere.

For the *E*/*I* balance of miniature frequency, the main effects of training (*F*_1,103_ = 0.75, *P* = 0.39) and interaction (*F*_1,103_ = 0.086, *P* = 0.77) were not significant (Fig. 4*O*), but right side of CA1 neurons exhibited higher E/I balance of the frequency than left side (*F*_1,103_ = 4.865, *P* = 0.03). The results suggest that CA1 neurons receive more excitatory inputs in the right hemisphere than in the left hemisphere, providing a synaptic evidence of laterality. Meanwhile, the training did not affect the balance of mEPSC versus mIPSC frequency, suggesting the balance of the number of excitatory versus inhibitory synapses onto ventral CA1 neurons.

### Self-Entropy of the Frequency in Ventral CA1 Neurons

Although the recorded neurons exhibited different self-entropy each other, self-entropy in the mEPSC frequency did not exhibit a significant main effect of training (*F*_1,103_ = 0.055, *P* = 0.82), laterality (*F*_1,103_ = 2.838, *P* = 0.095), or interaction (*F*_1,103_ = 0.147, *P* = 0.70, Fig. 4*M*). Similarly, self-entropy in the mIPSC frequency did not exhibit a significant main effect of training (*F*_1,103_ = 0.519, *P* = 0.47), laterality (*F*_1,103_ = 0.538, *P* = 0.47), or interaction (*F*_1,103_ = 0.342, *P* = 0.56, Fig. 4*M*). Thus, the training affected neither self-entropy regardless of the hemispheres. The Kernel analysis further visualized the density distribution (Fig. 4*N*). The average levels of self-entropy were 16.8 ± 2.7 bits (untrained) and 16.3 ± 1.7 bits (IA trained) per single CA1 neuron (Fig. 4*P*).

### The Number of Postsynaptic AMPA Receptor Channels

**Figure 5.**
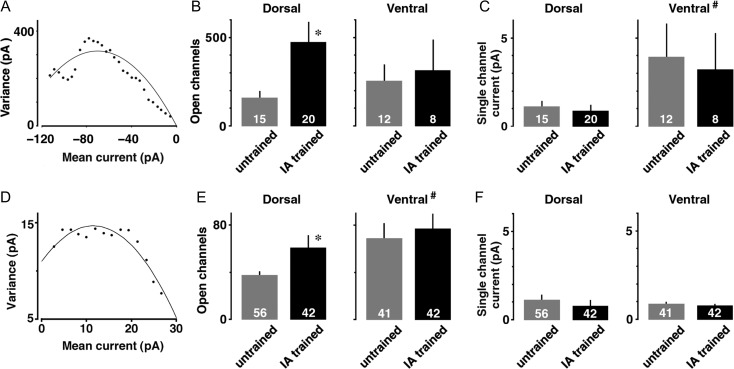
Estimated number of open channels and single channel current. (*A*) An example of nonstationary fluctuation analysis for AMPA receptor current. (*B*) Mean number of open Na^+^ channels at dorsal and ventral CA1 synapses. IA training significantly increased the number of open channels only at dorsal synapses. (*C*) Ventral synapses exhibited greater single-channel current than dorsal synapses, although the training did not affect the current. (*D*) An example of nonstationary fluctuation analysis for GABA_A_ receptor current. (*E*) Mean number of open Cl^−^ channels at dorsal and ventral CA1 synapses. IA training significantly increased the number of open channels only at dorsal synapses. Ventral GABAergic synapses possessed more channels than dorsal synapses. (*F*) Neither training nor CA1 region affected the single-channel current. The number of cells in each group is shown at the bottom of each bar. Error bars indicate + SEM. *P < 0.05 versus untrained. ^#^*P* < 0.05 versus dorsal.

To examine whether IA alters the number of AMPA receptors, we used evoked EPSC responses to calculate the number of opening AMPA receptors at dorsal or ventral CA3–CA1 synapses using nonstationary fluctuation analysis (Fig. 5*A*). The number of open AMPA receptors was significantly larger in IA-trained rats than untrained rats at dorsal CA3–CA1 synapses (*t*_33_ = 2.28, *P* = 0.029), but not at ventral CA3–CA1 synapses (*t*_18_ = 0.32, *P* = 0.75, Fig. 5*B*). Although the single-channel current was significantly greater at ventral synapses than dorsal synapses (*F*_1,53_ = 6.02, *P* = 0.017), the training did not affect the single-channel current (Fig. 5*C*). These results suggest that the training promotes postsynaptic plasticity by increasing the number of AMPA receptor-channels at dorsal but not ventral CA3–CA1 synapses.

### The Number of Postsynaptic GABA_A_ Receptor Channels

To examine whether IA alters the number of GABA_A_ receptors, we used mIPSC responses to calculate the number of opening GABA_A_ receptors at dorsal or ventral CA1 synapses (Fig. 5*D*). The number of open GABA_A_ receptors was larger in IA-trained rats than untrained rats at dorsal CA1 synapses (*t*_96_ = 2.30, *P* = 0.024), but not at ventral CA1 synapses (*t*_81_ = 0.44, *P* = 0.66, Fig. 5*E*). Although ventral synapses possessed more Cl^−^ channels than dorsal synapses (*F*_1,179_*=* 6.532, *P =* 0.011), neither area nor the training affected the single-channel current (Fig. 5*F*). These results suggest that the training promotes postsynaptic plasticity by increasing the number of GABA_A_ receptor-channels at dorsal but not ventral CA1 synapses.

### Bilateral Microinjection of Plasticity Blockers

**Figure 6.**
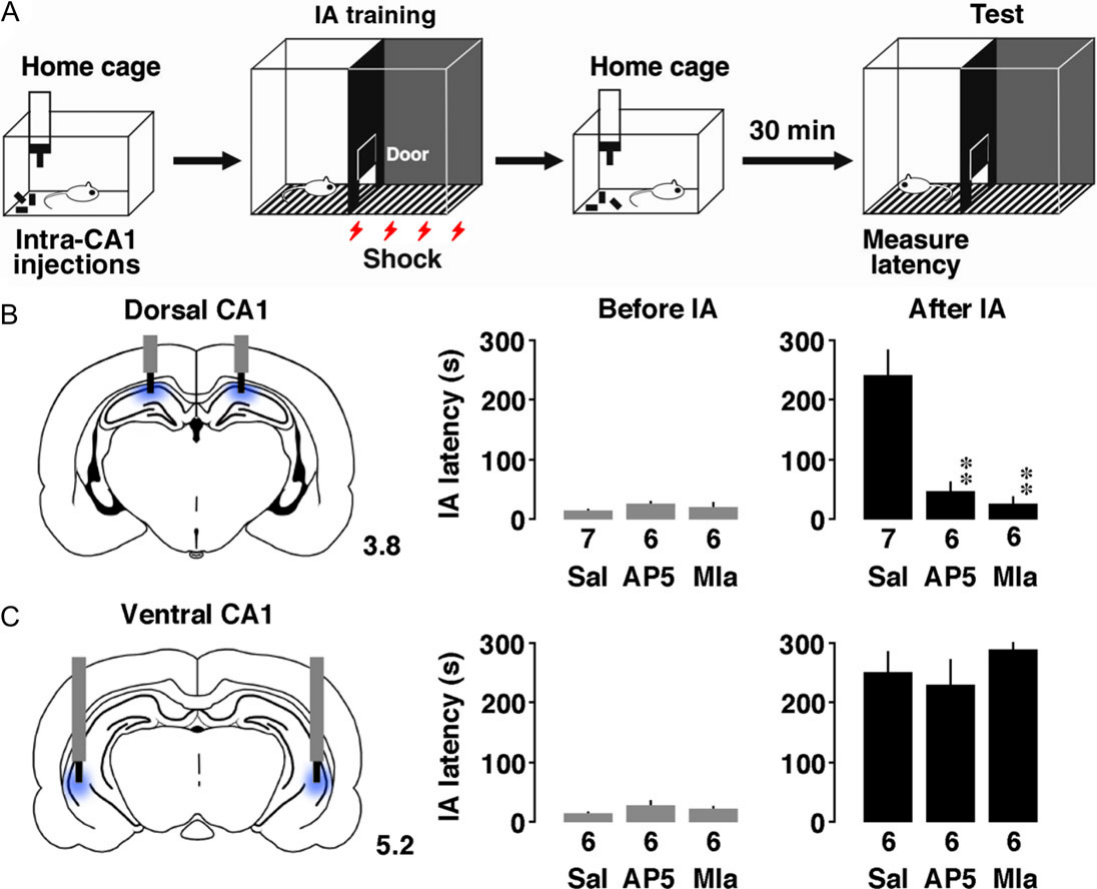
Intra-CA1 injection of plasticity blockers impairs the IA learning. (*A*) Experimental design of bilateral intra-CA1 injection and IA training. (*B*) Microinjection of AP5 or methyllycaconitine (Mla) into the dorsal CA1 impaired learning. (*C*) The injection into the ventral CA1 did not affect the performance. The number of rats in each group is shown at the bottom of each bar. Vertical gray and black bars indicate guide cannula and injector. The number indicates posterior coordinate from bregma. Error bars indicate + SEM. **P < 0.01 versus saline (Sal).

To examine the physiological role of plastic changes affecting the IA learning, saline, AP5, or Mla was bilaterally microinjected into the dorsal or ventral CA1 (Fig. 6*A*). In the dorsal CA1, both AP5 and Mla treated-rats showed shorter latency than saline-injected rats after the training (Fig. 6*B*, *F*_2,16_ = 16.411, *P* < 0.0001). Conversely in the ventral CA1, neither AP5 nor Mla treatment affected the latency (Fig. 6*C*, *F*_2,15_ = 0.773, *P* = 0.48).

## Discussion

Rat hippocampus is known to contain approximately 311 500 CA1 pyramidal neurons, receiving 13 059-28 697 CA3–CA1 synapses and up to 1 742 temporoammonic synapses from entorhinal cortex per single neuron (Bezaire and Soltesz 2013). Although both structural and functional heterogeneity are known in dorso/ventral or left /right CA1 neurons, the location of learning-induced synaptic plasticity has not been specified in the broad CA1 area. Here we found that the training increased AMPA receptor-mediated responses at dorsal CA3–CA1 synapses in both hemispheres, whereas ventral CA3–CA1 synapses did not show the plasticity in either hemisphere. The specified CA1 subfields of learning-induced plasticity provide a synaptic evidence of dorso/ventral heterogeneity at the synapse level.

Nonstationary fluctuation analysis further revealed evidence at a single channel level. We found the training significantly increased the postsynaptic number of open AMPA receptors at dorsal CA1 synapses, whereas the training did not affect the ventral CA1 synapses (Fig. 5*B*). By combining in vivo gene delivery and in vitro patch-clamp recordings, we previously demonstrated that contextual learning depends on synaptic delivery of GluA1-containing AMPA receptors at dorsal CA1 synapses at the molecular level (Mitsushima et al. 2011). The increase in the number of open channels without changes in the single-channel current further revealed learning-induced current changes at the AMPA receptor-mediated CA1 synapses.

The dorsal and ventral hippocampus play different roles based on distinct input and output connections (Swanson and Cowan 1977). Spatial and contextual memory appears to depend only on the dorsal hippocampus (Moser and Moser 1998; Strange et al. 2014), whereas ventral hippocampal lesions alter stress responses and emotional behavior (Henke 1990; Kjelstrup et al. 2002). In untrained rats, ventral CA1 neurons tended to show greater mEPSC frequency (mEPSC, *F*_1,98_ = 3.241, *P* = 0.075) and exhibited greater mIPSC frequency than dorsal neurons (mIPSC, *F*_1,98_ = 40.120, *P* < 0.0001; Fig. 4). This heterogeneity was already reported Millior et al. (2016), showing greater basal releases of both GABA and glutamate at ventral CA1 synapses. Moreover, as to the postsynaptic heterogeneity, ventral neurons showed greater mE(I)PSC amplitude than dorsal neurons (mEPSC, *F*_1,98_ = 11.324, *P* = 0.0011; mIPSC, *F*_1,98_ = 11.795, *P* = 0.0009; Fig. 3), suggesting greater postsynaptic AMPA/GABA_A_ current at ventral CA1 synapses in untrained rats. Finally, the greater single current of AMPA receptor (Fig. 5*C*) and more postsynaptic GABA_A_ channels (Fig. 5*E*) at ventral CA1 synapses may provide further evidence of dorso/ventral heterogeneity at the synapses.

Although laterality was not clear in our laboratory conditions, right side of CA1 exhibited more power of gamma oscillation and spine density than left side in rats reared in the spatially enriched conditions (Shinohara et al. 2013). In humans, patients with unilateral damage to the right hippocampus exhibit spatial memory deficits (Abrahams et al. 1996), whereas damage to the left hippocampus impairs verbal semantic representation (Richardson et al. 2004). In the present study, the right sides of synapses tended to have greater AMPA/NMDA ratios, mEPSC amplitudes, and self-entropy than the left side after training, though the laterality was not significant. Fibers through the ventral hippocampal commissure are known to connect bilateral CA1 (Amaral and Witter 1995; Kawakami et al. 2003), inducing high coherence of CA1 theta waves during running or REM sleep in the freely moving state (Patel et al. 2012). As unilateral CA1 blockade of AMPA receptor delivery (Mitsushima et al. 2011, 2013) fail to impair the IA learning, bilateral CA1 neurons may work together to compensate for impairment of the other in rats in normal laboratory conditions.

The question arises as to whether the synaptic strength contributes to memory. In regards to the excitatory synapses, contextual learning requires AMPA receptor delivery, as bilateral CA1 blockade of AMPA receptor delivery impairs learning (Mitsushima et al. 2011). Recently, Takemoto et al. further developed a technique to inactivate synaptic GluA1 AMPA receptors in vivo using chromophore-assisted light inactivation (CALI). Since optical inactivation of synaptic AMPA receptors successfully erased acquired-fear memory (Takemoto et al. 2017), newly delivered GluA1-containing AMPA receptors into the CA1 synapses seems to be required for contextual memory formation.

Learning also affects the GABA_A_ receptor-mediated inhibitory synapses, but the plasticity seems to be task-dependent at CA1 synapses (Cui et al. 2008; Mitsushima et al. 2013). Spatial learning presynaptically increases GABA release probability (Cui et al. 2008), whereas contextual learning postsynaptically strengthened GABA_A_ receptor-mediated inhibitory synapses (Mitsushima et al. 2013). Since optogenetic inactivation of somatostain-expressing interneurons in bilateral dorsal CA1 impaired the contextual fear learning, the learning seems to require the GABAergic inputs from somatostain-expressing interneurons at basal dendrites of dorsal CA1 neurons (Lovett-Barron et al. 2014). We further found a rapid phosphorylation of Ser^408–409^ GABA_A_ receptor β_3_ subunit within 5 min after the training (Sakimoto et al. 2017). Since the Ser^408–409^ phosphorylation is known to prevent clathrin adaptor protein 2-mediated GABA_A_ receptor internalization (Jovanovic et al. 2004; Lu et al. 2010; Petrini and Barberis 2014), the Ser^408–409^ phosphorylation may contribute to increase the number of GABA_A_ receptor channels (Fig. 5*E*) without changing Cl^−^ current per channel (Fig. 5*F*).

Sequential recording of mEPSCs and mIPSCs enables analysis of the strength of excitatory and inhibitory inputs in each neuron one-by-one. The increase in amplitude may indicate additional receptor delivery into the synapses, whereas the increase in frequency could result in an increase in the number of functional synapses or the probability of vesicle release from the presynaptic terminal (Kerchner and Nicoll 2008; Mitsushima et al. 2013). IA training clearly increased both mE(I)PSC amplitudes and frequencies in both hemispheres of dorsal CA1 neurons, whereas the training increased neither mE(I)PSC amplitudes nor frequencies in ventral CA1 neurons, regardless of the hemisphere. Moreover, the performance clearly impaired by the bilateral blockade of the plasticity in dorsal, but not ventral CA1 subfields, suggesting a specific role of the dorsal CA1 synapses for contextual learning (Fig. 6). Although the IA training failed to affect the synaptic plasticity in either hemisphere of ventral CA1 neurons, optogenetic cell body inhibition study revealed essential role of ventral CA1 neurons for social discrimination task (Okuyama et al. 2016).

Here we propose a new approach to quantify the learning-induced synaptic diversity in 4 CA1 subfields. The subfield-specific increase in self-entropy at dorsal, but not ventral, CA1 synapses in trained rats further provides an evidence of learning. Since bilateral blockade of the synaptic diversity clearly impaired the learning performance (Mitsushima et al. 2013, Ono and Mitsushima 2017), we hypothesized that the increased self-entropy may code a piece of experienced information after training. Present results not only confirmed previous findings, but also specified the subregion, suggesting a crucial role for IA learning (Fig. 6). Considering the total number of dorsal CA1 neurons (Andersen et al. 2006; Bezaire and Soltesz 2013), a possible increase in total self-entropy after IA training is estimated to be 12 550 000 bits. In any case, the quantification of the synaptic diversity in specific CA1 subfields would be a useful approach for diagnostic evaluation of cognitive disorders.

Synapses regulate cell firing according to the all-or-none principle (Kandel et al. 2013). If a neuron is considered an all-or-none device (Cannon 1922; Wiener 1961), one neuron can handle 1-bit of memory per clock cycle (log_2_ 2 = 1 bit; Hartley 1928). Based on the principle, computational theory proposed a role of the hippocampus as a kind of memory device (Marr 1977). We previously found a logarithmic relationship between the number of cells blocking plasticity and learning performance (Mitsushima et al. 2011), providing an evidence of binary processing of contextual information, such as what, where, or when. We hypothesized that the plasticity at excitatory/inhibitory synapses may change the binary processing of firing to encode memory. In support of this, real-time recordings of multiple unit activity of dorsal CA1 neurons further showed task-dependent diversified feature of ripple-like on/off firings (Ishikawa and Mitsushima 2016; Tomokage et al. 2018). Since selective elimination of the ripple-like events during post-training consolidation periods impairs the performance in dorsal hippocampus-dependent task (Girardeau et al. 2009), the events may contribute to code experienced information.

Synaptic dysfunction is well correlated with cognitive decline in Alzheimer’s disease (Terry et al. 1991). Amyloid beta (Aβ_42_) is well known as a major causative agent (Shankar et al. 2008; Querfurth and LaFerla 2010; Penzes et al. 2011; Sevigny et al. 2016), and long-term exposure to Aβ_42_ impairs the AMPA receptor trafficking by reducing synaptic distribution of CaMKII in cultured pyramidal neurons (Gu et al. 2009). As regard target molecule, hippocampal neurons that lack GluA3 were resistant against Aβ-mediated synaptic depression and spine loss, suggesting that Aβ initiates synaptic and memory deficits by removing GluA3-containing AMPA receptors from synapses (Reinders et al. 2016). On the other hand, as to the inhibitory synapses, Aβ_42_ specifically binds to nicotinic α_7_ receptors (Wang et al. 2000) promoting the learning-induced plasticity at the GABA_A_ receptor-mediated inhibitory synapses (Mitsushima et al. 2013; Townsend et al. 2016). Application of Aβ_42_, being known to block the nicotinic α_7_ receptor-mediated cholinergic response (Liu et al. 2001), quickly weakens GABA_A_ receptor-mediated synaptic currents via downregulation of GABA_A_ receptors (Ulrich 2015). Understanding the learning-induced plasticity in specific CA1 subfields as well as the quantification of synaptic diversity is necessary to diagnose the functional impairment in cognitive disorders, which may help to identify potential targets for therapeutic intervention.
